# Flexible Hydrophobic CFP@PDA@AuNPs Stripes for Highly Sensitive SERS Detection of Methylene Blue Residue

**DOI:** 10.3390/nano12132163

**Published:** 2022-06-23

**Authors:** Jinchen Dong, Tangchun Wang, Enze Xu, Feng Bai, Jun Liu, Zhiliang Zhang

**Affiliations:** 1 State Key Laboratory of Biobased Material and Green Papermaking, Qilu University of Technology (Shandong Academy of Sciences), Jinan 250353, China; dongjinchendr@163.com (J.D.); www2802608396@163.com (T.W.); xez15954319499@163.com (E.X.); bfeng0619@163.com (F.B.); 2 Faculty of Light Industry, Qilu University of Technology (Shandong Academy of Sciences), Jinan 250353, China

**Keywords:** hydrophobicity, gold nanoparticles, SERS, CFP@PDA@AuNPs stripes, methylene blue

## Abstract

Considering the inherent hydrophilic and porous nature of paper, the rapid absorption and diffusion of aqueous analyte solutions on paper-based SERS substrates may severely affect the Raman detection sensitivity and accuracy in the detection of target molecules. In this work, a series of hydrophobic CFP@PDA@AuNPs stripes were obtained through in situ synthesizing of gold nanoparticles (AuNPs) on a polydopamine (PDA)-decorated cellulose filter paper (CFP) and functionalized with perfluorodecanethiol (PFDT). When the SERS performance of the substrates was examined using 4-ATP, the hydrophobic CFP@PDA@AuNPs substrate showed superior sensitivity, reproducibility and stability due to the hydrophobic enrichment effect, with the detection limit decreasing to 10^−9^ M and the enhancement factor as high as 2.55 × 10^7^. More importantly, it was feasible to apply the hydrophobic paper substrate as an excellent SERS sensor to detect methylene blue (MB) residues in lake water in a highly sensitive manner. The lowest detectable limit of MB was 100 nM, and it showed a low relatively standard deviation (RSD) value of 5.28%. Hydrophobic CFP@PDA@AuNPs stripes may serve as excellent sensors for target molecule detection and have tremendous potential in food security, and environmental and chemical detection.

## 1. Introduction

As one of the important heterocyclic aromatic compounds containing a thiazine ring, methylene blue (MB) is widely used as an antibacterial agent to treat fish diseases, such as red mouth, saprolegniasis and fish scale disease, in the aquaculture and transportation fields [1,2]. However, excessive use of MB may cause serious vomiting, shock and tetraplegia in humans [3,4]. As a result, the use of MB to treat aquatic product disease in the European Union and the United States is banned; therefore, the need for a rapid and accurate detection of MB is fairly critical. At present, spectrophotometric, liquid chromatographic, photoacoustic, capillary electrophoresis and electrochemical methods are used as primary methods to detect methylene blue content [5,6]. Unfortunately, these approaches require expensive instruments, are time-consuming and not conducive to rapid detection of mass samples, meaning that they are not appropriate to execute rapid and effective MB detection.

Surface-enhanced Raman spectroscopy (SERS) represents a powerful and attractive analytical detection spectroscopy technique that can drastically boost the Raman signal of target molecules attached to or near the surface of noble metal nanoparticles [7]. The primary enhancement mechanism of SERS can be explained by the locally enhanced electromagnetic (EM) field that occurs near the metal nanostructures on account of localized surface plasmon resonance (LSPR) [8]. The intensity and frequency of LSPR are closely related to the incident light wavelength, the nanostructure of the substrate and the surrounding dielectric layer. Therefore, by controlling the morphology, size and spacing of nanoparticles, as well as their self-assembly, the best enhancement effect of the SERS substrate at the desired wavelength could be obtained [9]. SERS has a unique spectral fingerprint due to its vibrational characteristics based on the functional groups. It has been broadly applied in the food safety, environmental and biomedical fields as an analytical method with favorable sensitivity, non-invasiveness and rapidity [10,11,12]. In addition, a series of new techniques have recently emerged to collect Raman signals more efficiently, such as nanophotonic waveguides on photonic integrated circuits (PICs), which can serve as an alternative to conventional Raman microscopes without the need for a bulky and expensive instrument [13].

In recent years, paper-based SERS substrates have been gaining the attention of many researchers and have great advantages over traditional substrates due to their excellent flexibility and biocompatibility. In addition, the natural folded and porous structure facilitates the uniform loading of various nanometallic particles and the formation of abundant SERS “hotspots” [14]. A number of methods for the construction of paper-based SERS substrates that can achieve good SERS effects have been reported, such as solution impregnation [15], in situ growth [16], inkjet printing [17], vapor deposition [18] and the screen printing method [19]. Despite SERS substrates showing good sensitivity and reproducibility in the above methods, however, due to the inherent hydrophilic and porous nature of paper, the analyte solution is usually randomly distributed on the substrate surface, which leads to large variations in SERS signal intensity and severely reduces the sensitivity and homogeneity of SERS detection [20]. At the same time, it remains a big challenge to construct controllable paper-based SERS hotspot substrates and achieve a sensitive and reproducible Raman signal in an effective and rapid manner.

**Figure 1:**
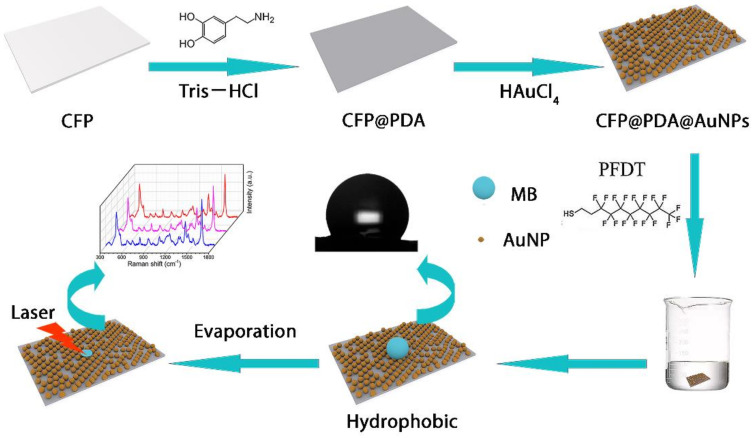
The schematic diagram of hydrophobic CFP@PDA@AuNPs SERS substrates for the highly sensitive detection of target molecules.

To address the above issues, this study proposed an effective approach to the construction of a low-cost and environmentally friendly hydrophobic paper-based SERS substrate with good sensitivity, reproducibility and stability. As shown Figure 1, cellulose filter paper (CFP) was firstly immersed into a dopamine solution to formalize polydopamine (PDA) layers on the CFP surface via self-polymerization of DA in a weak alkaline environment. Next, AuNPs were grown directly upon the CFP@PDA surface due to the in situ reduction ability of PDA, and CFP@PDA@AuNPs SERS stripes with hierarchical nanostructures were successfully synthesized. The CFP@PDA@AuNPs stripes were then hydrophobically modified with perfluorodecanethiol and achieved an optimal hydrophobic state with a contact angle of 132°. This would allow the CFP@PDA@AuNPs stripes to overcome the “analyte diffusion limit” on the SERS substrate surface at low concentrations and obtain an ideal detection sensitivity. When the performance of the SERS substrates was examined using 4-ATP, the SERS results demonstrated a limit of detection (LOD) of 10^−9^ M. More critically, residual methylene blue in lake water was also detected, and the detection limit of methylene blue was found to be 100 nM with R^2^ = 0.9704 and showed excellent point-to-point and sample-to-sample signal reproducibility. These results indicate that the developed hydrophobic CFP@PDA@AuNPs substrates may serve as sensitive sensors for rapidly detecting hazardous residues as well as having great potential in the food safety, environmental and biomedical fields.

## 2. Materials and Methods

### 2.1. Chemicals and Materials

1H, 1H, 2H, 2H-perfluorodecanethiol (PFDT), dopamine hydrochloride, 4-aminothiophenol (4-ATP) and tris(hydroxymethyl) aminomethane were obtained from Sigma-Aldrich. Ethanol and acetone were obtained from Kemiou Chemical Reagent Co., Ltd. (Tianjin, China). Methylene blue (MB) was supplied by Sinopharm Chemical Reagent Co. (Shanghai, China). Gold chloride trihydrate (HAuCl_4_·4H_2_O) was supplied by SiYu Chemical Technology Co. (Shanghai, China). The ultrapure water (18.2 MΩ) used throughout the experiment was produced by a Milli-Q system.

### 2.2. Fabrication of CFP@PDA@AuNPs SERS Substrates

Firstly, the small CFP sheets (1.2 × 0.6 cm^2^) were cleaned using deionized water and ethanol alternately with ultrasonication. Then, the CFP stripes

Were dipped into a DA solution (tris buffer, pH = 8.5) at 1 mg/mL concentration and magnetically stirred for 24 h to enable DA polymerization onto the CFP surface [21]. The obtained CFP@PDA stripes were subsequently rinsed off under deionized water before being dried at 50 °C. In the process of DA self-polymerization, the color of the CFP stripes changed from white to dark gray due to the oxidation of the catechol group of dopamine generating a large amount of melanin. (Figure S1a). Then, to in situ grow AuNPs on the surface of CFP@PDA stripes, the CFP@PDA stripes were soaked in a 1 mg/mL HAuCl_4_ solvent at pH = 5 for 24 h. The obtained CFP@PDA@AuNPs stripes were cleaned with deionized water before being dried.

### 2.3. Surface Hydrophobization on CFP@PDA@AuNPs SERS Substrate

As a kind of low-surface-energy material, PFDT could be applied to acquire a hydrophobic surface [22]. In this experiment, the CFP@PDA@AuNPs stripes were immersed in sealed solutions with different volume ratios of PFDT and ethanol for 12 h. Then, the obtained CFP@PDA@AuNPs stripes were rinsed three times with ethanol and dried at 40 °C to obtain the hydrophobic CFP@PDA@AuNPs SERS substrate.

### 2.4. SERS Detection of Hydrophobic CFP@PDA@AuNPs SERS Substrates

The SERS performance of hydrophobic CFP@PDA@AuNPs SERS substrates was assessed by selecting 4-ATP. First, a standard 4-ATP solution at a concentration of 10^−3^ M was prepared, after which it was sequentially diluted to a concentration range of 10^−5^–10^−9^ M with deionized water. Next, the 4-ATP solution required for each test was dropped onto the prepared hydrophobic substrate (Figure S1b), dried and subjected to Raman detection. For the detection of target analyte solutions, a range of different concentrations of MB solution was prepared by dissolving MB molecules with lake water, and the SERS detection procedure similar to that of the 4-ATP molecule was performed.

### 2.5. Morphology and Chemistry Characterization

A scanning electron microscope S-8220 (SEM, Hitachi, Japan) was used to characterize the surface structural and morphological aspects of all samples in the experiment. The samples’ crystal structures were studied using D8 Advance X-ray diffraction (XRD, Bruker, Germany). The elemental composition of the sample surface was analyzed using X-ray photoelectron spectroscopy (XPS) with an ESCALabXi+ from Thermo Electron. The elemental composition of the sample surface was analyzed using Al Kα (1486.6 eV) radiation as the X-ray source with a power of 150 W at a minimum of 10^−9^ Torr or lower pressure conditions. Raman spectra were collected on a RenishawinVia9 (Renishaw, UK) with 785 nm laser excitation. A 50× objective was used to focus on the sample surface, and the collection time was about 15 s. A Theta Flex optical contact angle meter (Biolin, China) with 4 μL water droplets was used to determine the water contact angle (WCA).

## 3. Results and Discussion

### 3.1. Morphological Structure and Compositional Analysis of CFP@PDA@AuNPs Substrates

**Figure 2:**
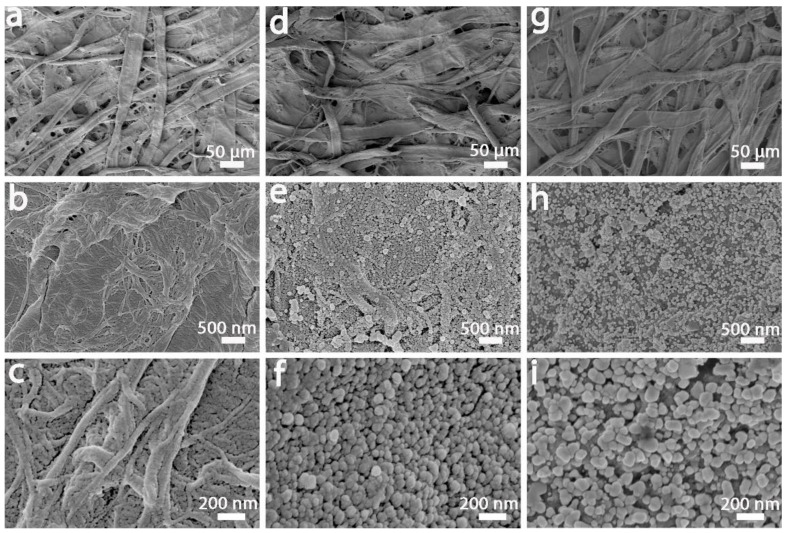
Representative SEM images of pure CFP (**a**–**c**), CFP@PDA (**d**–**f**) and CFP@PDA@AuNPs (**g**–**i**).

Morphology is very important to the SERS signal and is a key factor in maximizing the Raman response of the SERS substrate [23]. The morphology and nano-gaps of CFP@PDA@AuNPs stripes are strongly correlated with SERS property and determine the sensitivity and reliability from a true-world surface. To demonstrate the successful decoration of PDA and in situ growth of AuNPs on CFP surface, the morphological variation in CFP, CFP@PDA and CFP@PDA@AuNPs was determined by SEM. As illustrated in Figure 2a–c, the original CFP showed a three-dimensional mesh structure and consisted of cellulose fibers. In addition, the pristine CFP fibers exhibited a very smooth surface with no obvious nanostructures. After modification with dopamine (Figure 2d–f), the surface of CFP@PDA stripes became rougher, and some nanostructures appeared. It is possible that these nanostructures were formed due to the self-polymerization of PDA and were tightly adsorbed at the CFP@PDA stripe superficies. After the reaction of CFP@PDA in chloroauric acid solution, abundant AuNPs were in situ grown and deposited tightly on the substrate surface (Figure 2g–i). The size of AuNPs was about 80.1 ± 21.5 nm with spherical morphology according to the statistical results of SEM images, and the distance between these nanoparticles was small (Figure S2), which facilitated SERS hotspot formation and improved the performance of the SERS substrates.

**Figure 3:**
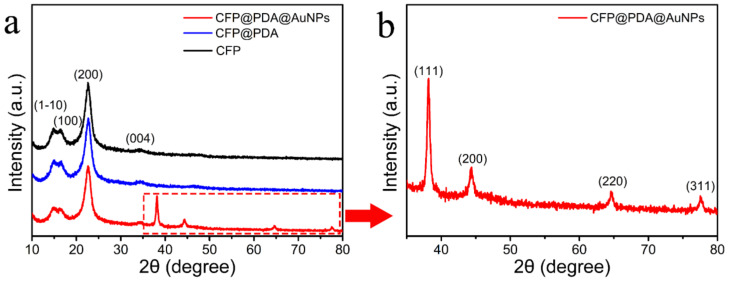
(**a**) XRD diffractogram for the pristine CFP, CFP@PDA and CFP@PDA@AuNPs. (**b**) High resolution XRD diffractogram of the CFP@PDA@AuNPs stripes.

The crystal structures of samples was monitored and analyzed using XRD during different stages of the experimental process. Figure 3a shows the XRD spectrum of the primordial CFP with four distinct diffraction peaks at 15.1°, 16.7°, 23.0° and 34.5°, attributed to the (1–10), (110), (200) and (004) facets of cellulose fibers, respectively [24]. It was also found that the XRD characteristic peaks of CFP@PDA stripes did not significantly change compared with the original CFP stripes, indicating that the generation of PDA would not change the crystal structure of CFP stripes. For the spectrum of CFP@PDA@AuNPs in Figure 3b, the diffraction peaks observed at 38.1°, 44.3°, 64.4° and 77.5° could be classified as the (111), (200), (220) and (311) crystal facets of AuNPs, respectively [25]. It was confirmed from the spectra that AuNPs were face-centered cubic structures on the surface of CFP@PDA@AuNPs.

**Figure 4:**
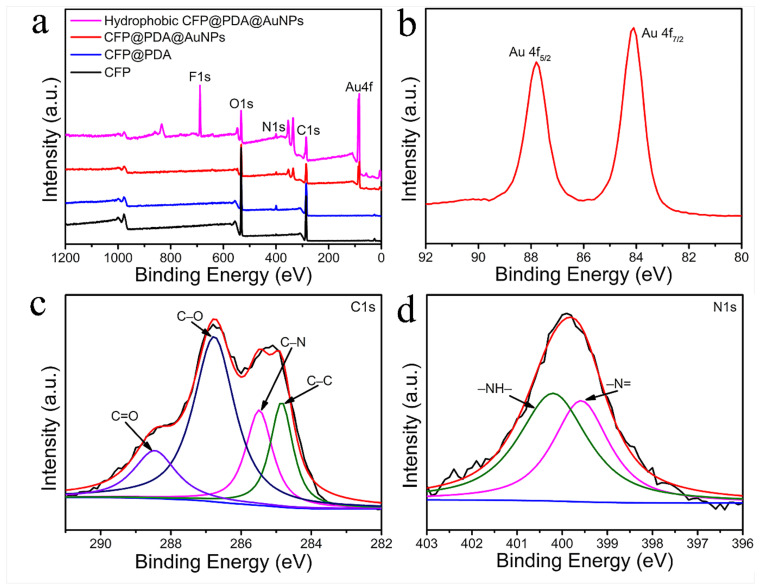
(**a**) XPS wide spectra of the original CFP, CFP@PDA, CFP@PDA@AuNPs and hydrophobic CFP@PDA@AuNPs. (**b**) High-resolution Au 4f spectrum of CFP@PDA@AuNPs. C1s (**c**) and N1s (**d**) peak fitting spectra of CFP@PDA.

The chemical composition and valence of CFP, CFP@PDA, CFP@PDA@AuNPs and hydrophobic CFP@PDA@AuNPs were carried out using XPS, and the related XPS spectra are presented in Figure 4. The XPS peaks at 285.95, 399.1 and 532.2 eV depicted in Figure 4a correspond to C1s, N1s and O1s, respectively. Moreover, it was found that the XPS spectrum of hydrophobic CFP@PDA@AuNPs showed a strong F1s peak at 688.8 eV compared to the other samples, which demonstrated the successful PFDT binding and hydrophobization of CFP@PDA@AuNPs stripes [26]. Compared with CFP and CFP@PDA, the XPS spectra of CFP@PDA@AuNPs and hydrophobic CFP@PDA@AuNPs showed distinct Au 4f characteristic photoemission peaks. In addition, the binding energies of 87.8 and 84.1 eV were observed for Au 4f_5/2_ and Au 4f_7/2_ in the narrow-scan XPS spectra of Au 4f (Figure 4b), which was consistent with the Au0 state [27,28]. 

In addition, Figure 4c,d show the core-level and fitted C1s and N1s spectra of CFP@PDA. The C1s and N1s spectra were fitted with the Thermo Avantage software, and the baseline correction was applied in smart mode. The curves were fitted based on the Gaussian–Lorentzian function, and the FWHM was controlled in the range of 0.3–1.7 eV. The C1s core-level XPS spectra (Figure 4c) were curve-fitted to four peaks (288.85, 286.75, 285.60, 284.8 eV) that were assigned to carbonyl group (C=O), C-O group, C-N group and C-C bond, respectively [29]. Likewise, the core-level XPS of N1s was classified into double peaks (399.70 and 400.35 eV), which represented the -N= and -NH- groups, respectively [30,31]. These analyses provide ample evidence for the effective modification of PDA. Interestingly, after the formation of AuNPs on the surface of CFP@PDA, the appearance of Au-N bonds was found when fitting their N1s high-resolution spectra (as shown in Figure S3) [32,33]. This may be due to the interaction between Au and the nitrogen-containing functional groups in the CFP@PDA structure, thus allowing the AuNPs to bind tightly to the CFP@PDA surface.

### 3.2. Optimization of the Hydrophobic Paper-Based SERS Substrates

The rapid absorption and diffusion of aqueous analyte solutions on paper-based SERS substrates may severely affect the Raman detection sensitivity and accuracy in the detection of target molecules. To address this issue, we employed a hydrophobic SERS-active surface and facilitated the generation of more SERS hotspots. Typically, two methods are used to formulate hydrophobic surfaces: the deposition of low-surface-energy substances on the surface to modify the chemical composition and the creation of micro- and nano-scale layered structures to enhance the rough surface structure [34,35]. As shown in Figure 2, there were plenty of micron-sized PDA bumps and AuNPs on the CFP@PDA@AuNPs surface, which constituted a micro- and nano-scale rough structure and was favorable for the formation of hydrophobic surfaces. To further improve the hydrophobicity of CFP@PDA@AuNPs, PFDT could be applied to acquire a hydrophobic surface.

**Figure 5:**
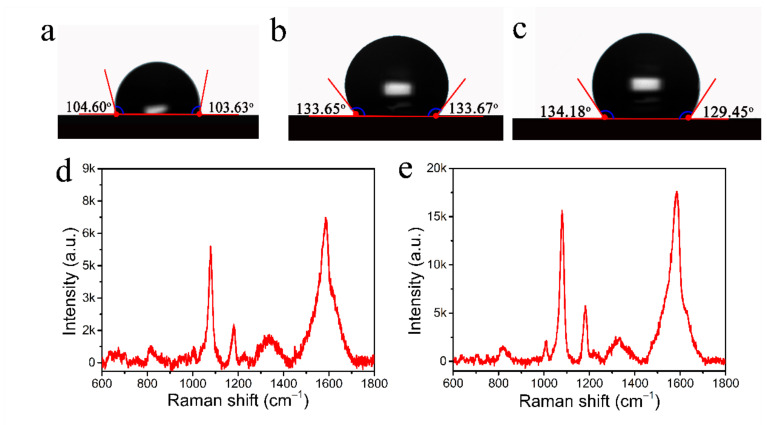
Variation in contact angle with the volume ratio of PFDT ethanol solution changed to (**a**) 1:1000 (**b**), 1:500 and (**c**) 1:250. (**d**,**e**) 4-ATP SERS spectra at 10^−7^ M concentration on the normal substrate and hydrophobic substrate.

To achieve an ideal hydrophobic state, the substrates were immersed in solutions with different PFDT volume ratios with ethanol (1:1000, 1:500, 1:250) for 12 h. As shown in Figure 5, the substrate exhibited low hydrophobicity with a contact angle of only about 103° in a volume ratio of 1:1000 PFDT solution. When the volume ratio was increased to 1:500, the hydrophobicity increased with a contact angle of about 132°, and as the volume ratio of the solution continued to increase to 1:250, the hydrophobicity increased minimally. Therefore, in subsequent experiments, we chose the PFDT ethanol solution with a volume ratio of 1:500 as the optimal condition. The SERS-enhanced signals of hydrophobic substrates versus normal substrates were also tested at a 10^−7^ M 4-ATP concentration. From Figure 5d,e, it can be seen that the signal intensity of the hydrophobic substrate was much higher than that of the normal substrate. All these results indicate that the hydrophobic modification of CFP@PDA@AuNPs could have an important influence on condensing the detected molecules for highly sensitive SERS detection.

### 3.3. SERS Performance of Hydrophobic CFP@PDA@AuNPs Substrates

**Figure 6:**
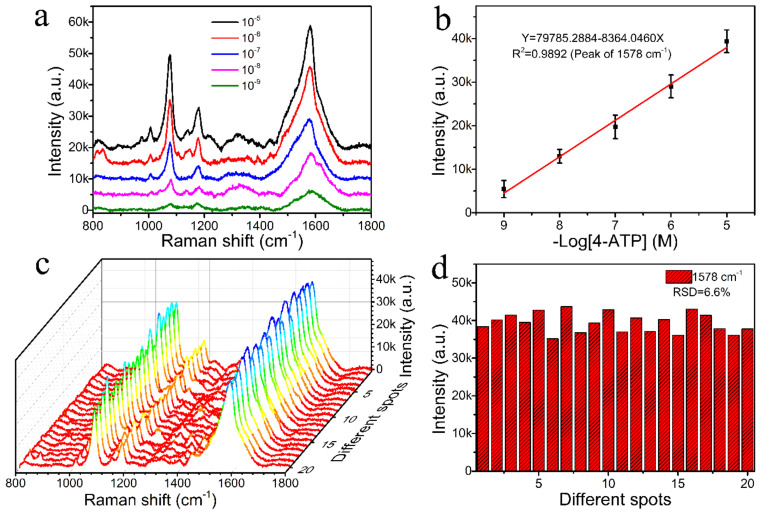
(**a**) SERS spectra obtained by employing 10^−5^ to 10^−9^ M of 4-ATP on the hydrophobic CFP@PDA@AuNPs substrate. (**b**) Linear fit diagram between the Raman intensity of the 4-ATP at 1075 cm^−1^ and the negative logarithm of the sample concentration. (**c**) Waterfall plots of 4-ATP (10^−5^ M) SERS signals recorded from 20 selected locations on the hydrophobic CFP@PDA@AuNPs substrate. (**d**) Corresponding histograms of peak intensity at 1075 cm^−1^ for the hydrophobic CFP@PDA@AuNPs substrate.

The classic probe molecule 4-ATP was first selected to measure the SERS sensitivity over a range of concentrations from 10^−5^ to 10^−9^ M. Figure 6a shows that the feature peaks of 4-ATP on the hydrophobic paper-based SERS substrates were mainly centered at 1004, 1075, 1140, 1178 and 1578 cm^−1^. The strong peaks at 1075 and 1578 cm^−1^ were allocated to the a_1_ vibrational modes (in-plane and in-phase modes); the peak at 1075 cm^−1^ was allocated to C-S stretching vibration; and the peak at 1578 cm^−1^ was allocated to C-C stretching vibrations [36,37]. The other peaks at 1004 cm^−1^, 1140 cm^−1^ and 1178 cm^−1^ were allocated to the b_2_ modes (in-plane and out-of-phase modes), and the two peaks at 1140 and 1178 cm^−1^ resulted from the bending of C-H modes [38,39].

It is also evident from Figure 6a that the Raman intensity sharply changed with the concentration of the detected 4-ATP. As the 4-ATP concentration was decreased to 10^−9^ M, the Raman signal remained distinctly identified, indicating that the hydrophobic CFP@PDA@AuNPs substrate processed excellent sensitivity to enable trace analyte detection. According to the formulas (Figure S4), the calculated enhancement factor of the hydrophobic CFP@PDA@AuNPs substrate was as high as 2.55 × 10^7^. Furthermore, as shown in Figure 6b, the SERS intensity at 1075 cm^−1^ exhibited an excellent linear fit to the concentration of 4-ATP, and the correlation coefficient was 0.9853. These results verify the high accuracy of the quantitative analysis of 4-ATP using a hydrophobic CFP@PDA@AuNPs substrate.

To evaluate the SERS reproducibility of the hydrophobic CFP@PDA@AuNPs stripes, the SERS signals were recorded at 20 different randomly selected locations, as shown in Figure 6c. The results indicate that the prepared substrates showed good SERS signal uniformity. Additional detailed analysis is presented in Figure 6d, and the relative standard deviation (RSD) of 9.2% was achieved for the SERS intensity at 1075 cm^−1^ from the different measurement points. This small RSD value confirmed that the hydrophobic CFP@PDA@AuNPs stripe could be applied as an excellent reproducible SERS sensor and achieve quantitative determination for target molecules in the real environment.

### 3.4. Application of Hydrophobic Substrates in Residual MB Detection

**Figure 7:**
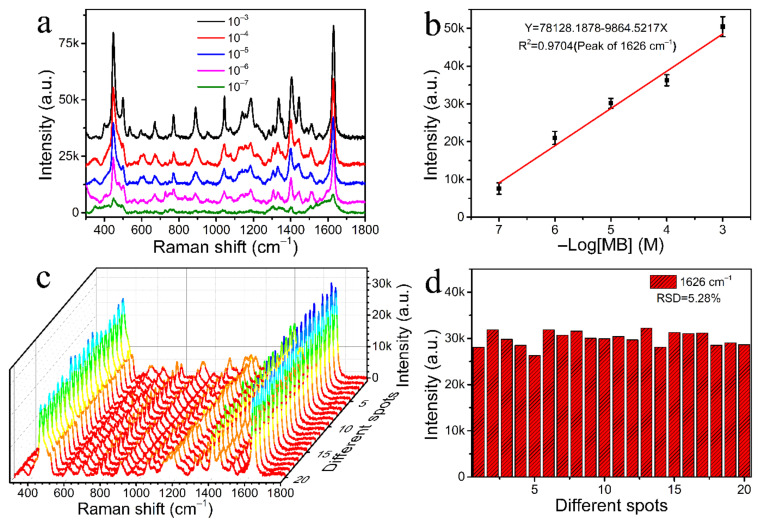
(**a**) SERS spectra obtained by employing 10^−3^ to 10^−7^ M of MB on the hydrophobic CFP@PDA@AuNPs substrates. (**b**) Linear fit diagram between the Raman intensity of MB at 1626 cm^−1^ and the negative logarithm of the sample concentration. (**c**) Waterfall graph of MB (10^−5^ M) SERS signals recorded from 20 selected sites on the hydrophobic CFP@PDA@AuNPs substrate. (**d**) Signal intensity map of MB collected at 1626 cm^−1^ from 20 selected sites on the same hydrophobic substrate.

To determine the actual SERS performance of hydrophobic CFP@PDA@AuNPs stripes, 15 μL of lake water solution at different concentrations of MB was subjected to SERS test by dropping it on the substrate surface and drying it at 40 °C. The Raman spectra of the lake water were tested before the experiment, and no interference with MB detection was observed (Figure S5). Figure 7a shows that the feature Raman peaks of the MB molecules mainly appeared at 1626, 1396, 1037, 770, 501 and 443 cm^−1^. The strong peak at 1626 cm^−1^ was ascribed to C-C ring stretching [40]. The double peaks displayed at 443 cm^−1^ and 501 cm^−1^ corresponded to C-N-C skeletal deformation [41]. The Raman band at 1396 cm^−1^ was attributed primarily to the stretching of the C-N bonds directly linked with the methyl groups of the molecule. The assignments of main characteristic peaks of MB are detailed in Table S1. A comparison of the theoretical and experimental MB peak positions is also shown in Table S1 [42]. It was found that there were some discrepancies between the calculated and observed values, which may be due to the interaction of MB molecules with the substrate surface and the effect of the vibrational modes, adsorption points and adsorption directions of the adsorbed molecules on the metal surface [43,44]. At the same time, the corresponding characteristic absorption peak could still be recognized even for MB concentrations as low as 10^−7^ M, which was below the maximum residue requirement of MB (0.4 ppm) [45]. This result suggests that this hydrophobic CFP@PDA@AuNPs substrate was able to rapidly and reliably detect MB. In addition, the SERS intensity of MB molecules on the hydrophobic CFP@PDA@AuNPs substrate was compared with that of the non-hydrophobic substrate, and it was found that the hydrophobic substrates showed higher intensity (as shown in Figure S6). This indicates that the hydrophobic modification of CFP@PDA@AuNPs could have an excellent concentrating effect and greatly improve SERS sensitivity.

The Raman feature scattering peak at 1626 cm^−1^ was utilized to assess the variation in SERS intensity as a function of MB concentration (Figure 7b), and a clear linear relationship was found between Raman intensity and MB concentration with a correlation coefficient of R^2^ = 0.9704. This result suggested that the hydrophobic CFP@PDA@AuNPs substrate may serve as an ideal SERS platform for rapid quantitative detection. With the purpose of testing its homogeneity, the SERS intensity of MB at 10^−5^ M was tested from 20 random points in the same sample. As illustrated in Figure 7c, the SERS substrates showed similar SERS peak patterns and almost identical intensity. The relative standard deviation (RSD) of these 20 different points at 1626 cm^−1^ was about 5.28% (Figure 7d), and these results demonstrate that the hydrophobic CFP@PDA@AuNPs substrates possessed good homogeneity.

**Figure 8:**
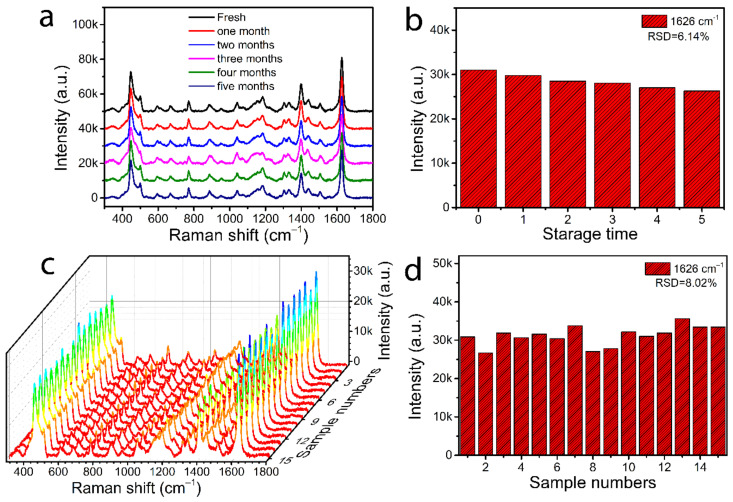
(**a**) SERS spectra obtained by employing 10^−5^ M MB on the fresh hydrophobic CFP@PDA@AuNPs and the variations with extended storage time. (**b**) Analysis of the intensity for the feature peak at 1626 cm^−1^ with increasing preservation time. (**c**) Waterfall graph of MB (10^−5^ M) SERS signals recorded from 15 various samples. (**d**) Corresponding histograms of peak intensity at 1626 cm^−1^ for 15 different samples.

In addition, reproducibility and temporal stability are two important factors for practical SERS detection applications. As illustrated in Figure 8a,b, the Raman spectra of MB molecules were tested with CFP@PDA@AuNPs substrates at a shelf-life longer than 5 months. As expected, the hydrophobic CFP@PDA@AuNPs substrate remained very stable up to 5 months of shelf-life. The Raman signal peak showed only a slight decrease at 1626 cm^−1^, and the RSD value was about 6.14%. As shown in Figure 8c,d, 15 different batches of hydrophobic CFP@PDA@AuNPs substrates were selected and used to perform respective SERS analysis. It was found that a similar signal intensity of MB molecules was obtained, and the calculated RSD value at 1626 cm^−1^ was only 8.02%, indicating good reproducibility and temporal stability of hydrophobic CFP@PDA@AuNPs substrates fabricated with our designed method.

## 4. Conclusions

In summary, a hydrophobic CFP@PDA@AuNPs SERS substrate was constructed based on the self-polymerization of DA, in situ growth of AuNPs and functionalization with PFDT on the CFP surface. Due to the hydrophobic enrichment effect, a detection limit as low as 10^−9^ M and enhancement factor as high as 2.55 × 10^7^ were achieved with the 4-ATP probe molecule, indicating superior sensitivity, reproducibility and stability. More importantly, the hydrophobic CFP@PDA@AuNPs could be directly used as an ideal platform for the detection of MB residues in lake water at a detection limit as low as 100 nM. This strategy could be used as an approach to synthesizing hydrophobic paper-based SERS substrates and has significant potential for applications involving food safety and environmental protection.

## Data Availability

The data presented in this study are available on request from the corresponding author.

## Supplementary Materials

The following are available online at https://www.mdpi.com/article/10.3390/nano12132163/s1, Figure S1. (a) Optical images of the original CFP, CFP@PDA and CFP@PDA@AuNPs from left to right. (b) Photograph of water droplets on the hydrophobic CFP@PDA@AuNPs stripe. Figure S2. (a) Results of particle size statistics of AuNPs. (b) SEM images of the CFP@PDA@AuNPs stripes. Figure S3. N1s peak fitting spectra of CFP@PDA@AuNPs. Figure S4. SERS spectra (black) for 15 μL 10^−9^ M 4-ATP on 36 mm^2^ of CFP@PDA@AuNPs and the Raman spectrum (red) for 10^−2^ M 4-ATP on a 110 mm^2^ silicon wafer. Table S1. Assignments of major peaks for methylene blue (MB). Figure S5. Raman spectra of lake water (red) and SERS spectra of 10^−5^ M MB lake water solution (blue). Figure S6. SERS spectra of (a) the normal CFP@PDA@AuNPs stripes and (b) hydrophobic CFP@PDA@AuNPs stripes at 10^−6^ M MB concentration.
