# Supplementary material for: Flexible Hydrophobic CFP@PDA@AuNPs Stripes for Highly Sensitive SERS Detection of Methylene Blue Residue
Source: Nanomaterials (Basel). 2022 Jun 23;12(13):2163. doi: 10.3390/nano12132163 (PMC9267967; doi:10.3390/nano12132163)
Supplement: Supplementary file 1 [file nanomaterials-12-02163-s001.zip › nanomaterials-1736455-supplementary.pdf]

nanomaterials

Supplementary Materials

Flexible Hydrophobic CFP@PDA@AuNPs Stripes for Highly Sensitive SERS Detection of Methylene Blue Residue

Jinchen Dong [1], Tangchun Wang [1], Enze Xu [1], Feng Bai [1], Jun Liu [2,*] and Zhiliang Zhang [1,*]

[1] State Key Laboratory of Biobased Material and Green Papermaking, Qilu University of Technology (Shandong Academy of Sciences), Jinan 250353, China; dongjinchendr@163.com (J.D.); www2802608396@163.com (T.W.); xez15954319499@163.com (E.X.); bfeng0619@163.com (F.B.)
[2] Faculty of Light Industry, Qilu University of Technology (Shandong Academy of Sciences), Jinan 250353, China
* Correspondence: liujun6621@126.com (J.L.); zhzhl@iccas.ac.cn (Z.Z.); Tel.: +86-0531-89631632 (Z.Z.)

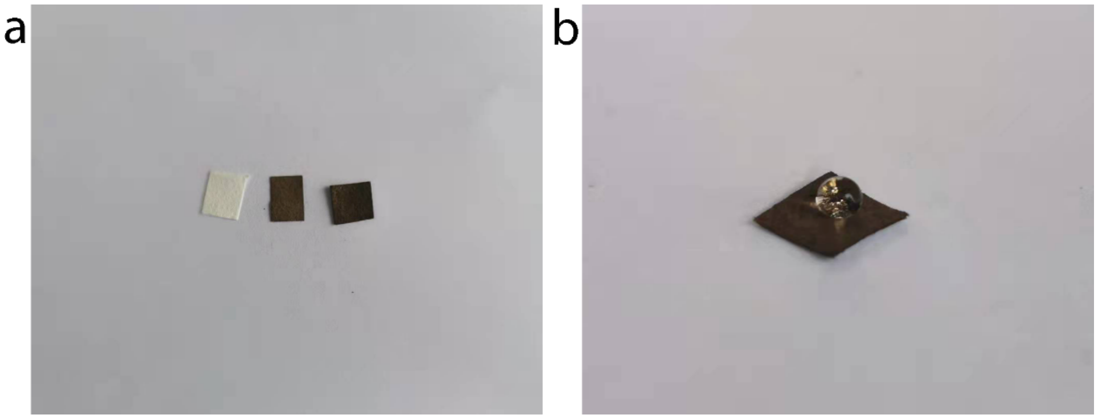

Figure S1. (**a**) Optical images of the original CFP, CFP@PDA and CFP@PDA@AuNPs from left to right. (**b**) Photograph of water droplets on the hydrophobic CFP@PDA@AuNPs stripe.

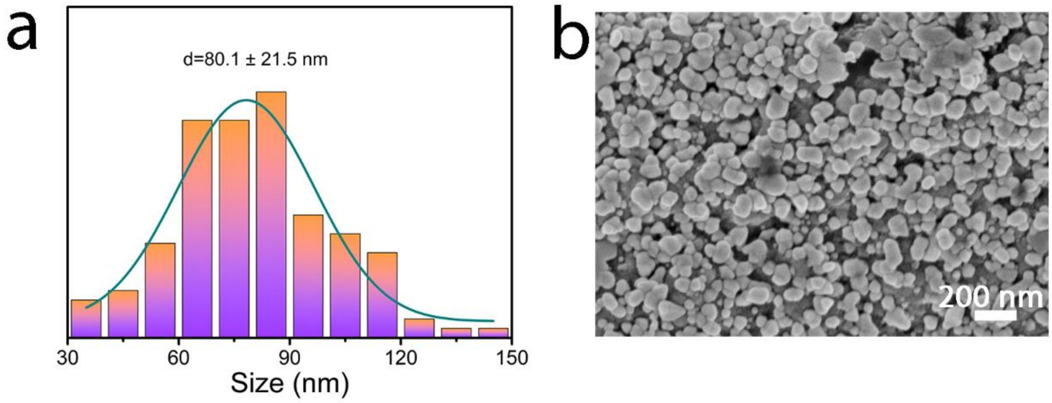

Figure S2. (**a**) Results of particle size statistics of AuNPs. (**b**) SEM images of the CFP@PDA@AuNPs stripes.

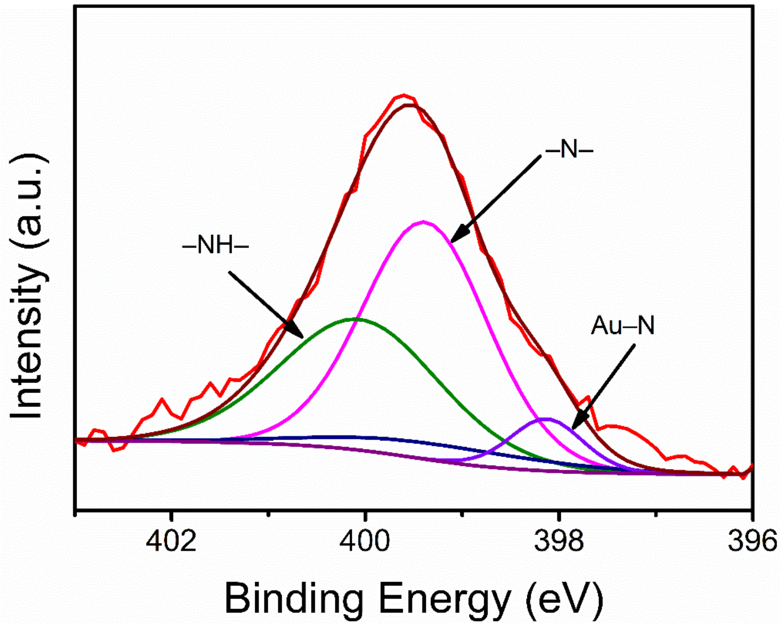

Figure S3. N1s peak fitting spectra of CFP@PDA@AuNPs.

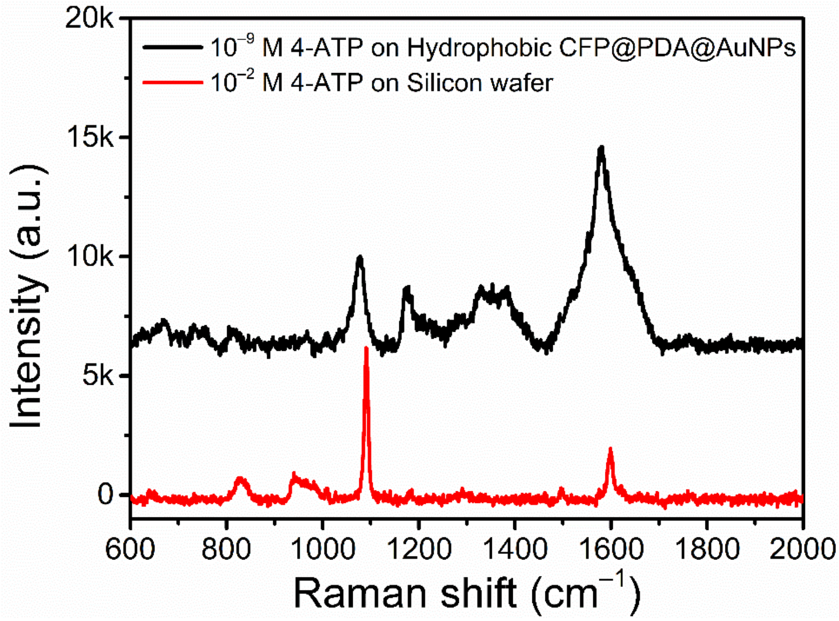

Figure S4. SERS spectra (black) for 15 μL 10^{-9} M 4-ATP on 36 mm^2 of CFP@PDA@AuNPs and the Raman spectrum (red) for 10^{-2} M 4-ATP on a 110 mm^2 silicon wafer.

Calculation of the Average Enhancement Factor (EF)

We used 4-ATP as probe molecule to estimate the averaged enhancement factor (EF). The enhancement factor (EF) could be computed to assess the SERS sensitivity of hydrophobic substrates according to the following equation:

$$EF = \frac{I_{SERS} N_{RS}}{I_{RS} N_{SERS}}$$

where I_{SERS} and I_{RS} represent the peak intensity of the 4-ATP molecule SERS signal and regular Raman spectrum at 1075 cm^{-1}, separately. N_{SERS} and N_{RS} are equal to the numbers of 4-ATP molecules on the respective substrates within the laser spots area, respectively. For simplification, N_{SERS} and could be calculated as follows:

$$N_{SERS} = n_{SERS} N_A = \frac{V_{SERS} C_{SERS}}{S_{SERS}} N_A$$

$$N_{RS} = n_{RS} N_A = \frac{V_{RS} C_{RS}}{S_{RS}} N_A$$

where n_{SERS} represent that a certain volume (V_{SERS}) and concentration (C_{SERS}) 4-ATP water solution was dispersed to an area of S_{SERS} on the hydrophobic CFP@PDA@AuNPs stripes; n_{RS}represent that a certain volume (V_{RS}) and concentration (C_{RS}) 4-ATP ethanol solution was dispersed to an area of S_{RS} at a clean Si wafer substrate. N_A represents Avogadro constant. Thus, the equation can be rewritten as follows:

$$EF = \frac{I_{SERS}}{I_{RS}} \cdot \frac{S_{SERS} V_{RS} C_{RS}}{S_{RS} V_{SERS} C_{SERS}}$$

In our experiments, 200 μL of 10 mM 4-ATP ethanol solution was dispersed to an area of about 110 mm^2 on Si wafer to record the Raman spectrum, and 15 μL of 1 nM 4-ATP ethanol solution was dispersed to on an area of about 36 mm^2 on the hydrophobic CFP@PDA@AuNPs stripes for SERS spectra. Taking 1075 cm^{-1} band for estimation, So the averaged EF for the band at 1075 cm^{-1} is calculated to be about 2.55×10^7.

Table S1. Assignments of major peaks for methylene blue (MB).

Calculated Raman	SERS (cm^{-1})	Band Assignments
445	443 (s)	α (C—N—C) AMG
498	501 (w)	α (C—N—C) AMG
594	595 (w)	α (C—N—C) AMG
669	669 (w)	α (C—C—C) Ring
763	770 (w)	ν(C—N) AMG; α(C—N—C) Ring
882	886 (w)	α (C—C—C) Ring
948	949 (w)	ϱ (CH2); β(CH)
1026	1037 (w)	β (CH); ν (C-S)
1394	1396 (m)	ν(C—N); ν(C—N) Ring; β(CH)
1445	1436 (w)	α(N—C—H) AMG; ν(C—C) Ring/ν(C—C) Ring
1633	1626 (s)	ν(C—C)/ν(C—N) Ring

s: strong; m: medium; w: weak peak intensity. ν: stretching; α, β: in-plane bending; ϱ: rocking. AMG: Attached to Methyl Group.

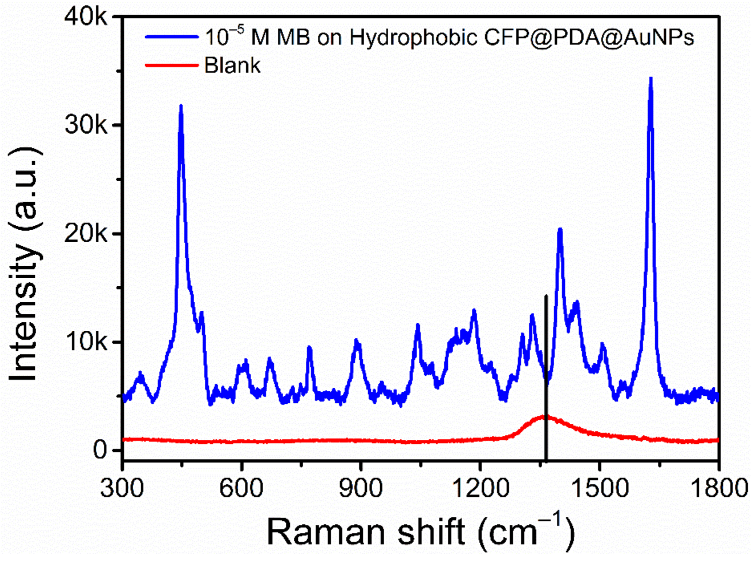

Figure S5. Raman spectra of lake water (red) and Raman spectra of 10^{-5} M MB lake water solution (blue).

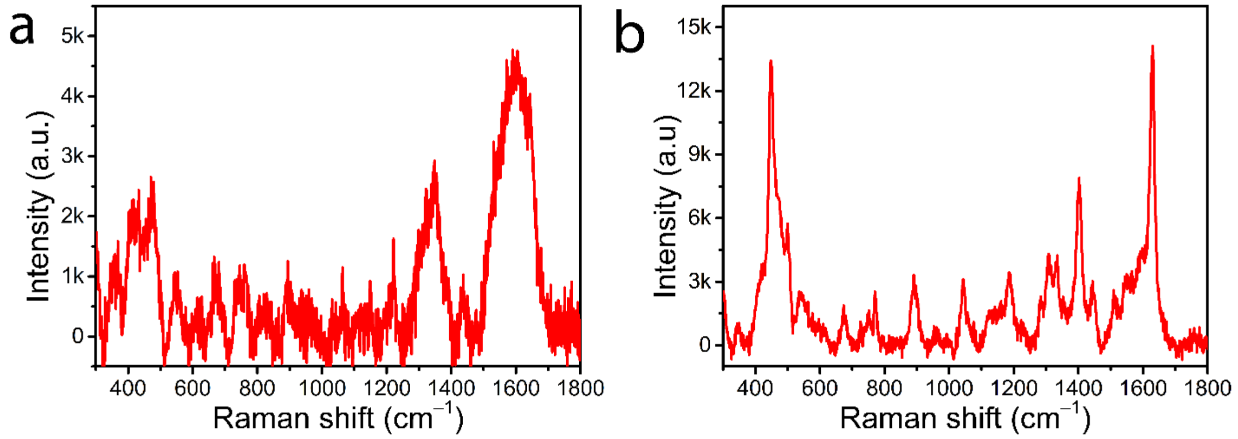

Figure S6. SERS spectra of on (**a**) the normal CFP@PDA@AuNPs stripes and (**b**) hydrophobic CFP@PDA@AuNPs stripes at 10^{-6} M MB concentration.
